# Supplementary material for: Higher visual areas act like domain-general filters with strong selectivity and functional specialization
Source: Nat Commun. 2026 Jun 12;17:7484. doi: 10.1038/s41467-026-73938-9 (PMC13408694; doi:10.1038/s41467-026-73938-9)
Supplement: Supplementary file 2 — Reporting Summary [file 41467_2026_73938_MOESM2_ESM.pdf]

nature portfolio

Corresponding author(s): Leila Wehbe

Last updated by author(s): Jun 5, 2025

Reporting Summary

Nature Portfolio wishes to improve the reproducibility of the work that we publish. This form provides structure for consistency and transparency in reporting. For further information on Nature Portfolio policies, see our Editorial Policies and the Editorial Policy Checklist.

Statistics

For all statistical analyses, confirm that the following items are present in the figure legend, table legend, main text, or Methods section.

n/a	Confirmed	
☐	☒	The exact sample size (*n*) for each experimental group/condition, given as a discrete number and unit of measurement
☐	☒	A statement on whether measurements were taken from distinct samples or whether the same sample was measured repeatedly
☐	☒	The statistical test(s) used AND whether they are one- or two-sided<br>*Only common tests should be described solely by name; describe more complex techniques in the Methods section.*
☐	☒	A description of all covariates tested
☐	☒	A description of any assumptions or corrections, such as tests of normality and adjustment for multiple comparisons
☐	☒	A full description of the statistical parameters including central tendency (e.g. means) or other basic estimates (e.g. regression coefficient) AND variation (e.g. standard deviation) or associated estimates of uncertainty (e.g. confidence intervals)
☐	☒	For null hypothesis testing, the test statistic (e.g. *F*, *t*, *r*) with confidence intervals, effect sizes, degrees of freedom and *P* value noted<br>*Give P values as exact values whenever suitable.*
☒	☐	For Bayesian analysis, information on the choice of priors and Markov chain Monte Carlo settings
☒	☐	For hierarchical and complex designs, identification of the appropriate level for tests and full reporting of outcomes
☐	☒	Estimates of effect sizes (e.g. Cohen's *d*, Pearson's *r*), indicating how they were calculated

Our web collection on statistics for biologists contains articles on many of the points above.

Software and code

Policy information about availability of computer code

Data collection	In the original study, Psychtoolbox 3.0.14, MATLAB 2018a and the Meadows web based research platforms were used.
Data analysis	We use python and pytorch for our analysis, as well as the network dissection package. Our full list of software and versions is available as part of our code.<br><br>The code used in this paper can be found at https://github.com/mkhosla-ucsd/response-optimized-modeling/

For manuscripts utilizing custom algorithms or software that are central to the research but not yet described in published literature, software must be made available to editors and reviewers. We strongly encourage code deposition in a community repository (e.g. GitHub). See the Nature Portfolio guidelines for submitting code & software for further information.

Data

Policy information about availability of data

All manuscripts must include a data availability statement. This statement should provide the following information, where applicable:

- Accession codes, unique identifiers, or web links for publicly available datasets
- A description of any restrictions on data availability
- For clinical datasets or third party data, please ensure that the statement adheres to our policy

We use the open 7T fMRI Natural Scenes Dataset (NSD):

Allen, E.J., St-Yves, G., Wu, Y., Breedlove, J.L., Prince, J.S., Dowdle, L.T., Nau, M., Caron, B., Pestilli, F., Charest, I., Hutchinson, J.B., Naselaris, T.*, Kay, K.* A massive 7T fMRI dataset to bridge cognitive neuroscience and artificial intelligence. Nature Neuroscience (2021).

The preprocessed dataset used in our study is available at https://drive.google.com/file/d/1FcY252RMhdh0E4U7BblfLsk74v74rkUZ/view . Source data are provided with this paper.

Research involving human participants, their data, or biological material

Policy information about studies with human participants or human data. See also policy information about sex, gender (identity/presentation), and sexual orientation and race, ethnicity and racism.

Reporting on sex and gender	The dataset has 8 participants (2 men and 6 women) and we do not look at sex or gender during our analyses. In fact they are not indicated in the dataset
Reporting on race, ethnicity, or other socially relevant groupings	We do not analyse or report on race, ethnicity, or other socially relevant groupings.
Population characteristics	The dataset has 8 participants: two males and six females; age range, 19–32 years
Recruitment	Participants were recruited by the University of Minnesota team according to the IRB approval from the University of Minnesota IRB.
Ethics oversight	The University of Minnesota IRB.

Note that full information on the approval of the study protocol must also be provided in the manuscript.

Field-specific reporting

Please select the one below that is the best fit for your research. If you are not sure, read the appropriate sections before making your selection.

[x] Life sciences   [ ] Behavioural & social sciences   [ ] Ecological, evolutionary & environmental sciences

For a reference copy of the document with all sections, see nature.com/documents/nr-reporting-summary-flat.pdf

Life sciences study design

All studies must disclose on these points even when the disclosure is negative.

Sample size	8 participants. Each participant viewed 10000 images that were used to train and test models of the visual system.
Data exclusions	None.
Replication	We train our models on 4 of the participants and can see that they generalize to held-out data from those participants and that they can easily be made to generalize to the remaining 4 participants.
Randomization	The 10000 images from the first 4 participants were split into training and test randomly. The models were randomly initialized multiple times.
Blinding	The participants were split into the two groups without knowledge of any specific characteristics.

Reporting for specific materials, systems and methods

We require information from authors about some types of materials, experimental systems and methods used in many studies. Here, indicate whether each material, system or method listed is relevant to your study. If you are not sure if a list item applies to your research, read the appropriate section before selecting a response.

Materials & experimental systems

n/a	Involved in the study
☒	☐ Antibodies
☒	☐ Eukaryotic cell lines
☒	☐ Palaeontology and archaeology
☒	☐ Animals and other organisms
☒	☐ Clinical data
☒	☐ Dual use research of concern
☒	☐ Plants

Methods

n/a	Involved in the study
☒	☐ ChIP-seq
☒	☐ Flow cytometry
☐	☒ MRI-based neuroimaging

Plants

Seed stocks: NA

Novel plant genotypes: NA

Authentication: NA

Magnetic resonance imaging

Experimental design

Design type: Event-related

Design specifications: Images were presented for 3 seconds, and repeated 3 times across the experiment. Each participant completed between 30 and 40 sessions, corresponding to an average of 38.5h of task based fMRI.

Behavioral performance measures: The participants were asked to indicate whether they had seen an image before, and their reaction times and button presses were recorded.

Acquisition

Imaging type(s): functional, anatomical

Field strength: 7T

Sequence & imaging parameters: Gradient-echo EPI at 1.8-mm isotropic resolution: slice thickness 1.8mm, slice gap 0mm, field-of-view 216mm (FE) × 216mm (PE), phase encode direction anterior-to-posterior, matrix size 120 × 120, TR = 1,600ms, TE = 22.0 ms, flip angle 62°, echo spacing 0.66ms, multi-band slice acceleration factor 3.

Area of acquisition: Whole brain

Diffusion MRI: ☐ Used ☒ Not used

Preprocessing

Preprocessing software: Allen et al. (2021) used a mixture of FreeSurfer, SPM, FSL, ANTs, MRTrix3 and their own code.

Normalization: We used the data in native space.

Normalization template: NA

Noise and artifact removal: We used the GLMdenoise version of the processed data, specifically: "betas_fithrf_GLMdenoise_RR"

Volume censoring: NA

Statistical modeling & inference

Model type and settings: We built our own fMRI response-optimized models to predict fMRI activity in response to images.

Effect(s) tested | We used the method of network dissection to interpret the selectivity of these models.

Specify type of analysis: ☐ Whole brain ☒ ROI-based ☐ Both

Anatomical location(s) | FFA, EBA, VWFA, RSC. These were obtained by the original dataset authors using a functional localizer.

Statistic type for inference (See Eklund et al. 2016) | We estimate prediction performance on held-out data.

Correction | NA

Models & analysis

n/a	Involved in the study
☒	☐ Functional and/or effective connectivity
☒	☐ Graph analysis
☐	☒ Multivariate modeling or predictive analysis

Multivariate modeling and predictive analysis | For each ROI, we train a convolutional neural network with a shared base and a readout component for each voxel to predict voxel activity to thousands of natural images.
