# Supplementary material for: Higher visual areas act like domain-general filters with strong selectivity and functional specialization
Source: Nat Commun. 2026 Jun 12;17:7484. doi: 10.1038/s41467-026-73938-9 (PMC13408694; doi:10.1038/s41467-026-73938-9)
Supplement: Supplementary file 3 — Transparent Peer Review file [file 41467_2026_73938_MOESM3_ESM.pdf]

nature portfolio

Peer Review File

Higher visual areas act like domain-general filters with strong selectivity and functional specialization

Corresponding Author: Dr Leila Wehbe

Parts of this Peer Review File have been redacted as indicated to remove third-party material.

This file contains all reviewer reports in order by version, followed by all author rebuttals in order by version.

Version 0:

Reviewer comments:

Reviewer #1

(Remarks to the Author)
This manuscript applies response-optimized convolutional neural networks for analysing the behaviour of functionally specialised higher order visual areas FFA, EBA, VWFA and RSC; leveraging a recently acquired public large-scale human fMRI dataset. As the authors note, sufficient human neuroimaging data for training modern neural networks directly (that is, without pretraining networks on computer vision tasks and -datasets and comparing the response profiles to brain data) has only been available for short time. Response-optimization methods have also explored in electrophysiology in macaques and rodents, which however is usually limited to early visual system areas like V1. The authors focus on human higher order processing areas instead.

The writing of this manuscript is clear and refreshingly thoughtful. I and several colleagues have already read the paper as a preprint, and my colleagues agreed with me that its novel analyses and discussions are inspiring.

Due to the novelty of the approach, the authors had to come up with several new analysis angles for response-optimized models. I could learn new ideas and techniques from this paper, for instance the surprisingly low data requirements of response-optimized models, the impact of training data deprivation (and discussion thereof), the IoU analysis for model interpretability, and that we might indeed know all relevant visual categories higher order areas respond to.

Furthermore it is positioning and contrasting the ideas behind leveraging the new datasets for response-optimization in a well-structured and insightful way, discussing the differences between various approaches. This alone makes it a very useful paper to the field.

The impact of papers following similar directions of leveraging novel massive datasets will expand beyond the visual system - since the researchers are investigating methods of copying aspects of information processing from a human brain system into modern neural networks.

This being said, I see the following points of improvement:

* The literature embedding is lacking a bit. The paper's approach and findings should be better contrasted to references (31-33), which the authors merely mention – without further discussion the previous work and embedding their own in the ongoing discourse – as related papers. They however all study convolutional architectures that were response-optimized in a similar way on large datasets, also including (yet not systematically surveying like the authors of this paper) higher order visual areas.

* The same goes for the discussion of the image synthesis results (69, 70, 24). Due to the interest into this topic there should be more emphasis on contrasting and comparing the results, as the prior-less ones presented in this paper are quite different from all other findings. It would be good to contrast these results more thoroughly to the results of the recent GAN prior-based results in humans by Gu 2022 [2] (on the same NSD data) and Murty 2021 [3] at least.

* The base architecture should be described by 2-3 sentences in the main text as the architecture is not methodologically irrelevant to assess the results – also given that the task-optimized baseline is the (potentially much more expressive / powerful) AlexNet architecture.

* Much of the research field comparing brain activity to convolutional neural network activations (or, to any representation used in the encoding framework) has silently agreed on using correlations for model comparison. For readers unfamiliar with the response-optimization ideas the almost-equal correlations between response-optimized and task-optimized in Figure 1B could raise some eyebrows, so this warrants added discussion. The widely discussed paper by Bowers et al. 2022 [1] could be referred to here as it is doing in-depth critical discussion about blindly using correlations for assessing deep nets as models of the visual system.

* Clarity could be improved in the main text description of the IoU method ("Network dissection reveals selectivity, tolerance and clutter-invariance"). (Overall the writing is very good, and this section is indeed an outlier in terms of comprehensibleness.)

* The models seem to behave quite a bit differently during the visual diet omissions, particularly in terms of the maximizing image synthesis. Could you discuss further what might be going on here?

* Could the choice of rotation-invariant networks have influenced the synthesized maximizing images? (I think this choice is comprehensible, also evident from Figure S5.)

* The paper presents response-optimization as being hypothesis-neutral approach, but it does not acknowledge that architectural choices (convolutional layers, rotation-invariance, etc.) do form a hypothesis which (as evident from the author's own supplementary analyses) also influences results. I would generally agree that response-optimization is hypothesis-neutral in contrast to task-optimization for the reasons the authors lay out, but we are still squeezing all this data into a convolutional neural network. I think the authors should acknowledge this influence in a potentially widely-read paper on the idea of response-optimization.

[1] Bowers, J. S., Malhotra, G., Dujmović, M., Montero, M. L., Tsvetkov, C., Biscione, V.& Blything , R. (2022). Deep problems with neural network models of human vision. Behavioral and Brain Sciences, 1-74.

[2] Zijin Gu, Keith Wakefield Jamison, Meenakshi Khosla, Emily J Allen, Yihan Wu, Thomas Naselaris, Kendrick Kay, Mert R Sabuncu, and Amy Kuceyeski. Neurogen: Activation optimized image synthesis for discovery neuroscience. NeuroImage, 247:118812, 2022.

[3] N. Apurva Ratan Murty, Pouya Bashivan, Alex Abate, James J. DiCarlo, and Nancy Kanwisher. Computational models of category-selective brain regions enable high- throughput tests of selectivity. Nature Communications, 12(1):1–14, 2021.

Reviewer #2

(Remarks to the Author)
The authors train artificial neural networks to predict voxel activity. These predictive models show selectivity as it typically seen in the human brain, such as face selectivity. This face selectivity even emerges if the training images did not include faces.

The authors performed a lot of detailed analyses. The methods and the findings are very relevant for the human visual neuroscience community. Nevertheless, I am not sure which truly new and important insights are offered compared to what is already in the literature.

In particular, although the details of the approach are very different, there are a lot of similarities with other recent papers that train neural networks to capture the selectivity in regions of interest, including Murty/Kanwisher in Nature Communications & Li/Bonner (Biorxiv). There are obvious differences (e.g., regions vs voxels; regions predefined through category selectivity vs voxels in general), but the general conclusion that such predictive models can be developed and capture a lot of aspects of cortical selectivity and function is very similar, as is the demonstration that such fitted models can then be used to make other predictions (generalization).

Also the observation that unconstrained, data-driven methods find selectivity for a limited set of categories (last sentence of abstract) is not very surprising. Other data-driven methods have suggested the same, see e.g. several articles by Lashkari and Vul (NeuroImage; J. Neurophysiol.).

One aspect of the data that I find particularly striking is the observation that there is high selectivity for the preferred category even in the absence of experience with the preferred category. This is consistent with recent single-unit data in monkeys by Vinken et al. ("The neural code for 'face cells' is not face specific"). However, in contrast to the research in monkeys, the current research does not give more insight into the features that are responsible for this generalization. This finding might be present because the neural selectivity incorporates the features of the typical category images. The extent to which this is a novel and impactful datapoint depends on more information on why this is found.

Many different features can be involved, which makes it difficult to test this exhaustively. I'll give one example, which will

also make clear what type of analyses might be relevant. Ohayon & Tsao (2012, Neuron) checked the importance of contrast features in face perception. As one part of their experiments, they worked with nonface images and analyzed how many contrast features they shared with a face template. A simple image subtraction of all non-face images with few contrast features from all non-face images with many contrast features resulted in a template that was very much face-like. This is striking because no face image was involved. I am not saying that the authors should check contrast features. However, the same analysis could already give some insights. In the nonface (face-deprived) experiment, the authors could take the (pixel-wise) average of all the nonface images that elicit strong responses and substract the average all the nonface images that elicit weak responses. How does the difference image look like? There are probably more nonlinear variants of this approach that could give insights into other features that are not defined in pixel space. There are some similarities between this approach and the methods behind Fig. 4 (which could maybe be repeated for the deprivation experiments), but I believe that more is needed. For the methods of Fig. 4 the way that the maximally exciting inputs look like depends very much upon the regularization methods that are used.

Smaller considerations:

1) can the authors show that the results in Fig. 4 are robust to changes in the many parameters that can be varied in this approach?
2) If I understand correctly, then Fig. 6C-D show the predicted responses to 'no persons' images on the left and to 'persons' on the right. Here the model did not see persons during training, but still the analyses in Fig. 6A-B suggest that the preferred concepts were still the same (e.g., head). Based on that finding in Fig. 6A-B, I would expect that the plots in Fig. 6C-D should show an overall higher response to the 'persons' images on the right than to the 'no persons' images on the left. The change in Y-axis interval complicates this comparison, but overall this prediction does not seem to be confirmed. Can the authors explain this?

Reviewer #3

(Remarks to the Author)
Khosla and Wehbe used deep neural network models (DNN) to analyze and interpret voxel-level activity in human higher visual areas of the 7T fMRI Natural Scenes Dataset (NSD). The models predicted quite accurately on how voxels responded to complex scenes and generalized quite rapidly to new subjects for several higher visual area ROIs, namely the fusiform face area (FFA), the extrastriate body area (EBA), the visual word form area (VWFA), and the retrosplenial cortex (RSC). More interestingly, analyzing these models provided provocative insights to interpret activity of these visual areas. The authors therefore concluded that their approach of using response-optimized DNN complements previous hypothesis-driven studies of the visual cortex, suggesting human higher visual areas may act like filters for selectively processing face, body, text, and place.

Although the manuscript describes a technology intensive work, it is in general well-written. There are several points I am hoping the authors could address to further strengthen it:

1. The selection of regions of interests (ROI) based approach. The main part of the current manuscript focuses on the FFA, EBA, VWFA and RSC. While the authors highlight that their approach was supposedly hypothesis-neutral, selecting these ROIs evidently relied heavily on hypotheses. Although I understand the last subsection of Results on pg. 16-17 and Figure 8 was more genuinely hypothesis-neutral (and I do think this part is more interesting), why did the authors not start from analyzing the whole ventral visual cortex and elaborate on this part? Alternatively, what about focusing on Brodmann areas or anatomically defined ROIs?

A couple of minor points relate to this issue: a) there are other face areas such as the occipital face area and the superior temporal sulcus, other scene processing area such as the parahippocampal place area, that are also well-known from previous research. The motivation of choosing the current ROIs but not other known category areas should be provided. b) Providing further method details on how exactly the ROIs were defined in the current study would be helpful.

2. Relationship of activity between different voxels. The brain is obviously not organized by voxels as fMRI voxel is an arbitrary parcel that contains population of neurons. Based on the definition of functionally localized ROI such as FFA, EBA, VWFA and RSC, activity of voxels within an ROI should relatively correlate rather than be independent from each other. Not surprisingly, the results (Figure 5) to a large extent confirmed this point while also revealing different activity profiles between voxels within a same ROI – as the authors state that tuning differences between voxels in the same ROI were captured. However, it is not clear what this result means to understand how populations of neurons are clustered/organized based on their functions. Would it be possible the interrelationship among neurons between different voxels confound current interpretation of the models? For example, presumably if voxel size is too big, the models would fail to capture more category tuning information, but how do we know the current voxel size and shape are appropriate? On the other hand, it is well-known that category information can be decoded from multi-voxel activity patterns. Is it plausible to use response-optimized models to analyze activity patterns across multi-voxels? If so, might more category selectivity beyond face, body, text and food be found?

I am also curious about relationship of voxel activity between different ROIs. If I understand correctly, through the present approach face-selective voxels were also found in the VWFA and EBA. Were text-selective voxels also found in the FFA, EBA, and RSC etc.? Are tuning functions of these voxels similar to tuning functions of the voxels selective to the conjectured preferred category of their corresponding ROIs, i.e., face-selective voxels in the FFA, text-selective voxels in the VWFA etc.? I think that exploring further on this issue and elaborating on what could be interpret from this cross-ROI analysis would be very informative.

3. Reconstructed maximally exciting images. Honestly, to me they do not look like faces, texts…. Is it possible to run a quick fMRI experiment on a few subjects comparing how these images may actually activate the FFA, EBA, VWFA and RSC vs. how faces, bodies, texts and places activate these ROIs?

4. Results shown in Figure 7 are confusing or perhaps misleading. Apparently, according to these results, the RSC is better at face recognition and letter classification than layout classification; the EBA is better at face recognition and letter classification than pose prediction. Especially for the EBA, face recognition performance and letter classification performance are comparable or even better than ImageNet, but prose prediction is worse than ImageNet. Should we still call it a "body area" instead of a face area or a word form area? This is highly counterintuitive and, I think, warrants further discussions.

Other relatively minor issues:

1. Many previous studies have shown idiosyncrasies of category selectivity in the ventral visual stream. For example, the FFA differs greatly across individuals as face recognition ability varies from prosopagnosia to super recognizer. Individual expertise experiences have also been argued to implicate selectivity of the FFA. Given that data from only 8 subjects were tested in the present study, external validity of the current findings warrants further discussions.

2. Activity in the ventral visual stream is actually not fully invariant to low-level features. In addition to the well-known inversion effect of face and text processing as well as the contrast-negation effect of face recognition, high contrast and large stimulus size in general lead to greater activity in the ventral visual stream than low contrast and small stimulus size. On the other hand, intrinsic cues also activate the ventral visual stream (e.g., Cox, Meyers & Sinha, 2004). Can models in the present study speak to these complicated response properties of the ventral visual stream, or are they beyond the scope of the present study?

3. While the VWFA is normally only found in the left hemisphere, the FFA is often found in both hemispheres but larger in the right than in the left. It is not clear whether the present study noted the hemispherical lateralization, or simply pooled data from both hemispheres.

Version 1:

Reviewer comments:

Reviewer #1

(Remarks to the Author)
The authors have thoroughly addressed all of the points raised in my review, and I am satisfied with the revisions made. The manuscript is now suitable for publication. I would also like to thank the authors for their detailed and thoughtful responses in the rebuttal letter.

(Remarks on code availability)
The code is clearly written and commented. The authors also provide demos with limited data that can easily be used. A README file and examples in Jupyter notebooks are also provided. The code can be used for understanding what has been done and for reproduction, and become the basis for similar projects.

Reviewer #2

(Remarks to the Author)
I thank the authors for the thorough discussion of the points that I brought up. I have sympathy for many of their points. This is an interesting study with a lot of innovation at the level of using brain data to train computational models.

Nevertheless, I am concerned that novelty for the understanding of the neural basis of object recognition and categorization is still not well characterized and several neuroscience-related findings in the literature are mostly ignored despite being very similar. Maybe my previous comments were at times too conceptual and as such not always probing for specific actions, so I'll give two very specific suggestions that are already implied by my previous review.

First, a better discussion of the differences with Ratan Murty et al (2021) is needed. This study already showed the relevance of computational models for high-throughput tests (and alternatives for fMRI experiments), and many of the messages are the same. The submitting authors point to the use of task-trained models in that study as the major difference with their own training on brain data. This is not as absolute though. Ratan Murty also trained the classifier with brain data, with the difference being in where the input features came from. However, in addition, Ratan Murty showed good performance with

relatively generic feature layers (late convolutional layers), and for models with very different task objectives some of which are very remote from the investigated categories (e.g., using training on scenes with Places365 to give features for a classifier trained on FFA responses). So as soon as high-level features are present, it works. Findings like Murty's make me less surprised by the findings in the submitted manuscript. I can see the interest from a modelling perspective (look, we seem to have enough data to train on the brain data per se!), but the novelty from a neuroscience and cognitive point of view is less prominent to me once I consider what Murty et al found.

Second, the authors should refer to the work of Vinken et al. (2023, Science Advances) that came to very similar conclusions in terms of higher visual responses acting like "domain-general filters". The similarity in the main conclusions is striking. A deep discussion of this convergence is needed. Given that I mentioned this paper very clearly in my previous review, and the authors also cite that paper when replying to my comments, I am surprised that the Vinken et al paper is even not mentioned at all in the revised version. (And no, I am not an author of that paper.)

As another lingering concern, an important part of the paper concerns the training with and without the preferred category being present. Figure 6 and the summary in the manuscript emphasize how well this works, but the summaries should retain the important finding that the correspondence with the preferred concept is apparently going down from 0.13 to 0.08, so halfway to the threshold of 0.04 that the authors set themselves throughout the paper. This is a major reduction of selectivity, and should be mentioned in e.g. Abstract and Discussion. At the very very least, the relevant sentence in the abstract should read "this selectivity is MOSTLY maintained", and summaries in e.g. Discussion should mention this reduction. Also, in the figures, it seems important to plot the IoU to the preferred category of the face/body-deprived models next to the IoU of the non-deprived models.

Note also that the stimulus set does not include images that are optimized for ROIs like FFA and EBA. The example "most activating images" in Fig. 3 make this very obvious. FFA is most activated by close-up images of faces, not by the images shown here (also Ratan Murty showed this very clearly). As a consequence, the fitted models might miss important aspects of the selectivity of such ROIs, and it is very much conceivable that these are the selectivity properties that are most strongly affected by the presence/absence of faces in the image set. Or, to put it in the context of the text that the authors added on p. 15, would you expect a particularly high response in your FFA model for close-up faces, and would you predict this to be the same with face deprivation? Such out-of-distribution tests seem very important here, considering that they implicate the sort of images that other studies have shown to be optimal for FFA. The lack of such images in your stimulus set is worth discussing.

Finally, it now occurs to me that the authors selected ROIs/domains for the deprivation test. I can see why you would do that for RSC given that the preferred category is probably difficult to remove completely from natural images, but I don't find good arguments for why one would not include VWFA in this test. There were not so many billboards in the images according to the authors, so this test should be very easy to implement? Or did the authors try and it failed?

(Remarks on code availability)

Reviewer #3

(Remarks to the Author)
First of all, I would like to thank the authors to take such a long time to address my previous concerns. I think the manuscript has been strengthened to a large extent. However, I remain concerned about the following points.

#3.1
I understand that to train a network for the entire visual cortex would not be possible. As a matter of fact, there has never been any clear definition boundary of where exactly 'visual cortex' may include. However, the current overall framing of the manuscript heavily relies on and indeed still stresses on 'hypothesis-neutral'. And I agree that the manuscript should have been a lot stronger if the approach could really be hypothesis-neutral. As I suggested previously, using anatomically defined ROIs would make the current study a lot closer to a hypothesis-neutral approach. Note that the so called *ventral* visual cortex involves a mixture of anatomical and functional concepts, therefore may not be ideal. But subsampling subsections of the inferior temporal cortex that can be anatomically defined should be feasible and more like hypothesis-neutral for functional category selectivity.

#3.5
Using GAN is a good idea! And I agree the new set of generated images are much easier to interpret. However, it is not clear what brain mechanisms may correspond to GANs that can be added on to the current part of visual processing. I think the authors do make a decent stab of explaining why this greatly helps the visualization of voxel selectivity (p.21-22) but equally I think that potential readers may still get confused on how the brain could incorporate generative mechanisms or GAN-based priors versus diffusion model priors for visual category selectivity. Elaborating theoretical possibilities or flag this as a theoretical limitation in the Discussion section would be greatly helpful.

#3.6
I understand that classification accuracy should not be compared across tasks. However, Figure 7 is still confusing. Comparing within the same task, performances of the other three ROIs than the category selective ROI often vary, sometimes apparently significantly differ (e.g., for letter classification and for body pose classification). Use ImageNet

performances (blue triangles) as a benchmark to compare within each task may be a good idea. But this should be more explicitly described in the manuscript. Plus, text descriptions (p. 17) do not match Figure 7. The authors should proofread the manuscript more carefully.

(Remarks on code availability)

Version 2:

Reviewer comments:

Reviewer #2

(Remarks to the Author)
The authors have addressed all my remaining suggestions. I congratulate them with this very nice paper.

(Remarks on code availability)

Reviewer #3

(Remarks to the Author)
Again, first of all I thank the authors for taking so long to address our concerns. But I have to say this process has taken way too long. I had forgotten many details and had been quite confused with how the authors addressed my second round comments, until I found my first round comments to the original submission over two years ago.

In my first round comments, I highlighted the results shown in Figure 8 (Figure 2 in the response letter of this round), and suggested the authors to elaborate on this part of their study. I suggested this because, as I pointed out in the first round, this part was genuinely hypothesis-neutral and thus most interesting. Unfortunately, the authors apparently did not take the suggestion as an opportunity to strengthen the paper in that round of their revision. They however highlighted Figure 8 only in the response letter of this round to address my concerns. Again, my suggestion is to work on this part as potentially stronger evidence to support their claim of hypothesis-neutral. It should not be about convincing me or anyone as a reviewer. I just think that highlighting and elaborating on this part will strengthen the paper and benefit potential readers and thus making the paper potentially more impactful.

(Remarks on code availability)

Open Access This Peer Review File is licensed under a Creative Commons Attribution 4.0 International License, which permits use, sharing, adaptation, distribution and reproduction in any medium or format, as long as you give appropriate

credit to the original author(s) and the source, provide a link to the Creative Commons license, and indicate if changes were made.
In cases where reviewers are anonymous, credit should be given to 'Anonymous Referee' and the source.
The images or other third party material in this Peer Review File are included in the article's Creative Commons license, unless indicated otherwise in a credit line to the material. If material is not included in the article's Creative Commons license and your intended use is not permitted by statutory regulation or exceeds the permitted use, you will need to obtain permission directly from the copyright holder.
To view a copy of this license, visit https://creativecommons.org/licenses/by/4.0/

Reply to the Reviewers

"Higher visual areas act like domain-general filters
with strong selectivity and functional specialization"
Meenakshi Khosla and Leila Wehbe
Nature Communications

Dear Editor,

We thank you for arranging the review and giving us an opportunity to improve our manuscript. We also thank the reviewers for their careful reading of our manuscript and their helpful comments. We have updated our manuscript to address all of the reviewers' comments. Below, we include the full text of the review with our response to all the questions raised by the reviewers in blue below each point, including all major changes made in the main manuscript (referenced below by section). We believe our response addresses all questions raised by the reviewers.

REVIEWER COMMENTS

Reviewer 1 (Remarks to the Author):

Reviewer #1, comment #1

This manuscript applies response-optimized convolutional neural networks for analysing the behaviour of functionally specialised higher order visual areas FFA, EBA, VWFA and RSC; leveraging a recently acquired public large-scale human fMRI dataset. As the authors note, sufficient human neuroimaging data for training modern neural networks directly (that is, without pretraining networks on computer vision tasks and datasets and comparing the response profiles to brain data) has only been available for short time. Response-optimization methods have also explored in electrophysiology in macaques and rodents, which however is usually limited to early visual system areas like V1. The authors focus on human higher order processing areas instead.

The writing of this manuscript is clear and refreshingly thoughtful. I and several colleagues have already read the paper as a preprint, and my colleagues agreed with me that its novel analyses and discussions are inspiring.

Due to the novelty of the approach, the authors had to come up with several new analysis angles for response-optimized models. I could learn new ideas and techniques from this paper, for instance the surprisingly low data requirements of response-optimized models, the impact of training data deprivation (and discussion thereof), the IoU analysis for model interpretability, and that we might indeed know all relevant visual categories higher order areas respond to.

Furthermore it is positioning and contrasting the ideas behind leveraging the new datasets for response-optimization in a well-structured and insightful way, discussing the differences between various approaches. This alone makes it a very useful paper to the field.

The impact of papers following similar directions of leveraging novel massive datasets will expand beyond the visual system - since the researchers are investigating methods of copying aspects of information processing from a human brain system into modern neural networks.

Our response #1.1

We thank the reviewer for their positive assessment. We address each of the concerns point-by-point and in detail below.

Reviewer #1, comment #2

** The literature embedding is lacking a bit. The paper's approach and findings should be better contrasted to references (31-33), which the authors merely mention – without further discussion the previous work and embedding their own in the ongoing discourse – as related papers. They however all study convolutional architectures that were response-optimized in a similar way on large datasets, also including (yet not systematically surveying like the authors of this paper) higher order visual areas.*

Our response #1.2

We now include an expanded literature review discussing these relevant papers in the *"Related studies"* section of the discussion. It now reads:

"In conjunction with our current research, recent studies [1–3] have demonstrated the competitive performance of the response-optimization approach when trained with extensive datasets, often comparable to state-of-the-art task-optimized models. Khosla et al.[3] adopt similar neural network architectures for modeling a series of areas along the ventral visual hierarchy, from the earliest stages of processing at V1 to intermediate stages V2-V4 and higher-order ventral-occipital (VO) and lateral-occipital (LO) regions and provide a strong quantitative validation of the response-optimization approach. Seeliger et al.[1] optimize parameterized models directly using responses from early visual areas during prolonged naturalistic stimulation and demonstrate the ability of trained models to predict the population receptive fields of these areas. St. Yves et al.[2] studied the early to intermediate visual cortex (V1-V4) and explored the brain hierarchy's role in training brain-optimized models and presented counter evidence against the hierarchical nature of neural representations. Our study differs from these works in several aspects:

- We extend beyond population receptive field estimation and employ a novel network dissection method to analyze the feature selectivity of units in trained networks, generating verbal descriptions of neural response properties. We extend beyond population receptive field estimation and employ a novel network dissection method to analyze the feature selectivity of units in trained networks, generating verbal descriptions of neural response properties.

- We showcase how response-optimized models can be used to tackle critical scientific questions about selective responses. Through visual deprivation experiments, we reveal how category-selectivity can be influenced by domain-general mechanisms.

- Our research uncovers a crucial link between emergent representations in response-optimized models and their functional roles. We demonstrate how the response-optimization approach yields models that excel at fine-grained perceptual tasks, providing empirical evidence for task specialization in distinct category-selective regions of interest (ROIs). Notably, this work marks the first assessment of the functional capabilities of response-optimized models, i.e., their generalization to behavioral tasks. Interestingly, our models' strong generalization ability sometimes surpasses the generalizability of even highly transferable representations from ImageNet-trained networks, yielding intriguing implications for computer vision models.

Collectively, these studies, along with our research, advocate for an alternative and complementary strategy to the prevailing task-optimized modeling framework for understanding neural representations."

Reviewer #1, comment #3

** The same goes for the discussion of the image synthesis results (69, 70, 24). Due to the interest into this topic there should be more emphasis on contrasting and comparing the results, as the prior-less ones presented in this paper are quite different from all other findings. It would be good to contrast these results more thoroughly to the results of the recent GAN prior-based results in humans by Gu 2022 [2] (on the same NSD data) and Murty 2021 [3] at least.*

Our response #1.3

We now include an expanded literature review discussing these relevant papers in the discussion section *"Visualization of voxel selectivity with image synthesis."*. This also refers to a new analysis we add in supplementary figure S10 and here in Figure 1. This analysis uses a GAN prior to generate the maximal exciting inputs for our response-optimized networks. It now reads:

"We perform an unconstrained optimization over input noise to discover the input that would result in maximal excitation of individual voxels, which we refer to as the maximally exciting inputs. It is important to note that by performing unconstrained optimization, we are not restricting the space of maximally activating inputs to the naturalistic domain; instead, we can let the optimization process evolve arbitrarily complex visual inputs. This is in contrast to prior approaches of modeling higher-order cortical activity, which rely on GAN-based priors for synthesizing maximally activating images[4,5] or more recently on diffusion models prios.[6] Such approaches use class-conditional GANs or diffusion models trained on large-scale datasets for image synthesis. They either optimize the image class first and then fine-tune the noise vector space,[5] optimize both jointly to maximally activate neural responses,[4] or use the gradient of the brain signal to directly guide the generation of new images.[6] Additionally, these studies synthesize maximally activating images at the region-level and not at a finer-grained voxel level.

For completeness, we perform a thorough comparison with some of these existing approaches for visualizing maximally activating inputs, particularly those of Ratan et al.[4] and Gu et al.[5] which employ GAN-based priors. However, instead of performing optimization with task-optimized models of brain activity, here we apply similar techniques to our response-optimized models of higher-order sensory regions. Our optimized images, depicted in Supplementary Fig. ß10, again highlight the strong and distinct category-selective tuning of these regions, aligning with their previously hypothesized selectivity. Notably, using GAN-based priors has made the optimized images more comprehensible, contrasting with outcomes from methods lacking naturalistic priors (as in Fig.4). However, we argue that both types of approaches are useful. The prior-free approach which we adopt in this manuscript can reveal the core visual forms that drive high-level category-selectivity in response-optimized models, even if the resulting images are harder to interpret. Consider that

visualization with a natural image prior may not always be truly faithful to the model. For instance, generative model priors might inherently favor complex visual features (such as the presence of natural object categories) and favor categories that are over-represented in the dataset used to train the prior, irrespective of the tuning properties of voxels; the content of their visualizations, thus, has a greater risk of being linked correlationally, and not causally, to brain activation.[7] As an example, Gu et al.[5] have reported the prevalence of dogs in the maximally activating images for FFA. These seemingly counter-intuitive findings could potentially be clarified by considering the characteristics of ImageNet, the dataset upon which most task-optimized encoding models are trained. ImageNet comprises 120 different dog breeds but lacks explicit categories for humans. In contrast, the prior-free MEI generation method we use here avoids such biases as it does not depend on pre-trained datasets."

Reviewer #1, comment #4

** The base architecture should be described by 2-3 sentences in the main text as the architecture is not methodologically irrelevant to assess the results – also given that the task-optimized baseline is the (potentially much more expressive / powerful) AlexNet architecture.*

Our response #1.4

We now describe the base convolutional architecture in the result section, section *"Accurate predictions on complex, cluttered scenes..."*. It now reads:

"We used a rotation-equivariant Convolutional Neural Network (CNN) architecture. This equivariant architectural design choice enables the model to learn identical features at multiple orientations and spatial locations, mimicking the response properties of neurons in early and intermediate visual areas, which are known to capture similar features like edges and curves but at different orientations and locations in the visual field.[8–11] This core "representational" network comprises four convolutional blocks, each with two layers and followed by an equivariant Average-Pooling operation. Each convolutional layer contains 48 feature sets where each feature set is extracted at 8 orientations and is followed by a ReLU non-linearity. We employed a linear *readout* model on top of this feature space to predict the responses of individual voxels in the ROIs. A linear readout on a shared feature space is further reminiscent of the often made assumption that the activity in one region in one individual can be related to the activity in the homologous region in another individual by a linear transform.[12] The linear readout was *factorized* into *spatial* and *feature* dimensions following popular methods for neural system identification in mouse visual cortex.[13] This factorization separates receptive field location (i.e., what portion of the visual space is the voxel most sensitive to?) from feature tuning (i.e. what features of the visual input is the voxel sensitive to?). Sharing the entire representational network across subjects, sharing convolutional filters weights across visual field locations (translation equivariance) and orientations (rotation equivariance) and a factorized readout jointly enable the sample-efficient training of the response-optimized models (see Appendix D)."

We further note that we also demonstrate the impact of architecture on model performance in the Supplementary (Figure S5) where we include the AlexNet baseline. Interestingly, the response-optimized model with the AlexNet architecture performs worse than the response-optimized model with the proposed rotationally-equivariant architecture. This might happen because the presence of useful inductive biases in the rotationally-equivariant architecture helps the model converge on a good generalizable solution with limited examples.

Reviewer #1, comment #5

** Much of the research field comparing brain activity to convolutional neural network activations (or, to any representation used in the encoding framework) has silently agreed on using correlations for model comparison. For readers unfamiliar with the response-optimization ideas the almost-equal correlations between response-optimized and task-optimized in Figure 1B could raise some eyebrows, so this warrants added discussion. The widely discussed paper by Bowers et al. 2022 [1] could be referred to here as it is doing in-depth critical discussion about blindly using correlations for assessing deep nets as models of the visual system.*

Our response #1.5

We agree with the reviewer's assessment of the limitations of correlation-based measures for assessing

[editorial note: figure redacted]

Figure 1: **Maximally exciting inputs obtained through optimization with GAN-based priors**. We perform constrained optimization using a class-conditional GAN (BigGAN) as the prior. The GAN is coupled with the response-optimized model of each target brain region and new stimuli are synthesized to maximize the mean predicted response across all voxels in the target region, following Murty et al. (2021) (A) and Gu et al. (2022) (B).

model-brain similarity in general. In order to add more clarity, we added additional comments to the ”Brain response predictivity” section of the discussion. In this paper, we go far beyond correlating a network that is trained on a task and brain activity (an approach that Bowers et al. 2022 argue is limited). We actually train networks exclusively to predict that very same brain activity. Correlation is only used as to verify that the models are appropriate and have enough accuracy, not to derive scientific conclusions. For our scientific conclusions, we interpret the *predictions* of these models using tests of increasing ”severity” (to borrow the term by Bowers et al. 2022) : we interpret the selectivity of those predictions, we show that selectivity arises even in the absence of the category of interest in training, and we show that a network that is trained to predict a brain region learns functional properties that allow it to better classify the category of interest than networks trained for other brain regions. That last test is reminiscent of the severe tests advocated for by Bowers et al. 2022, because we look at the functional properties of these response-optimized networks. We never correlate *representations*.

Reviewer #1, comment #6

** Clarity could be improved in the main text description of the IoU method ("Network dissection reveals selectivity, tolerance and clutter-invariance"). (Overall the writing is very good, and this section is indeed an outlier in terms of comprehensibleness.)*

Our response #1.6

Thank you, we have now edited the description in the section *"Network dissection reveals selectivity, tolerance and clutter-invariance"* of the results.

Reviewer #1, comment #7

** The models seem to behave quite a bit differently during the visual diet omissions, particularly in terms of the maximizing image synthesis. Could you discuss further what might be going on here?*

Our response #1.7

We note that the macro-scale behavior of the models in terms of their selectivity profiles remains the same even in the deprivation case. The deprived models, however, reveal the simpler featural selectivity that underlies high-level category selectivity of the respective ROIs.

Reviewer #1, comment #8

** Could the choice of rotation-invariant networks have influenced the synthesized maximizing images? (I think this choice is comprehensible, also evident from Figure S5.)*

Our response #1.8

We agree with the reviewer that the equivariant architecture might have affected the synthesized images, since it has affected prediction accuracy. We have added discussion in the result section, under *"Hypothesis-agnosticity reveals a limited set of categories for which selective responses are observed in the ventral visual cortex"*, where we also specify that while this choice is not entirely hypothesis-neutral, it remains neutral with respect to the visual tasks relevant to our high-level ROIs.

Reviewer #1, comment #9

** The paper presents response-optimization as being hypothesis-neutral approach, but it does not acknowledge that architectural choices (convolutional layers, rotation-invariance, etc.) do form a hypothesis which (as evident from the author's own supplementary analyses) also influences results. I would generally agree that response-optimization is hypothesis-neutral in contrast to task-optimization for the reasons the authors lay out, but we are still squeezing all this data into a convolutional neural network. I think the authors should acknowledge this influence in a potentially widely-read paper on the idea of response-optimization.*

Our response #1.9

Thank you for this comment. As in the previous comment, we agree with your astute observation that the response-optimization approach is not entirely hypothesis-neutral since it relies on a pre-specification

of the architecture, which may constrain the emergent representations and mechanisms. Within the same result section as above, we acknowledge this issue.

Reviewer 2 (Remarks to the Author):

Reviewer #2, comment #1

The authors train artificial neural networks to predict voxel activity. These predictive models show selectivity as it typically seen in the human brain, such as face selectivity. This face selectivity even emerges if the training images did not include faces.

The authors performed a lot of detailed analyses. The methods and the findings are very relevant for the human visual neuroscience community. Nevertheless, I am not sure which truly new and important insights are offered compared to what is already in the literature.

We thank the reviewer for their positive assessment. We have addressed each of the comments that were potentially critical point-by-point below

In particular, although the details of the approach are very different, there are a lot of similarities with other recent papers that train neural networks to capture the selectivity in regions of interest, including Murty/Kanwisher in Nature Communications & Li/Bonner (Biorxiv). There are obvious differences (e.g., regions vs voxels; regions predefined through category selectivity vs voxels in general), but the general conclusion that such predictive models can be developed and capture a lot of aspects of cortical selectivity and function is very similar, as is the demonstration that such fitted models can then be used to make other predictions (generalization).

Our response #2.1

We note that the neural networks in the cited references are not trained specifically to capture activity in regions of interest. Instead, their ability is heavily influenced by training on large semantically-labeled datasets, which are known to already contain units selective to a wide variety of semantic categories.[14,15] In contrast, the emergence of strong complex category selectivity in networks trained solely with supervision from brain responses provides much stronger empirical evidence of category selectivity. The response-optimization approach allows extraction of computational principles directly from brain data, presenting a promising alternative to the traditional task-optimization approach for building models of brain activity (which is strongly hypothesis-constrained). Further, as also noted by Reviewer 1, while such methods have been explored in the context of electrophysiology recordings in rodents, their application has been primarily constrained to modeling early visual areas. We have added discussion pertaining to these points in the *"Related studies"* section of the discussion. Our work provides a promising illustration that with the proliferation of large-scale neuroimaging datasets, this approach can even be applied to high-level perceptual areas, without sacrificing predictive accuracy (with even quantitative advantages in certain scenarios, as in generalization to novel subjects), providing a robust platform for testing computational theories.

We further emphasize that none of the previous works demonstrate functional (task-specific) differences between activity in these ROIs because they exploit networks trained on a general object categorization objective. In this work, we demonstrate that response-optimized models exhibit different fine-grained discrimination behaviors, providing empirical support for their functional specificity. While many studies suggest functional roles for these ROIs, direct evidence from brain activity or modeling has been limited. In macaques, while there is preliminary evidence that face-selective neural populations carry some information about face identity,[16] the evidence from human fMRI has been even more limited. Furthermore, comparisons between different task-optimized models fail to reveal any functional distinctions. For instance, deep neural networks trained on face individuation perform significantly worse than or on par with networks trained on object categorization in capturing representations in face-selective regions in the primate brain. Beyond FFA, studies comparing neural networks trained on diverse tasks consistently find that networks trained on object recognition are the best models of the entire visual stream (including dorsal and lateral streams), thus failing to reveal any functional differences between different brain regions. In contrast, our models show significant functional distinctions. For instance, we demonstrate that among the different response-optimized models, the model optimized for recapitulating activity in FFA best performs face individuation and the model optimized to predict VWFA activity performs best at letter discrimination, and so on. The observation that these complex visual capacities are realized spontaneously in neural networks optimized solely with neural constraints is noteworthy and, to out knowledge, has never been demonstrated before.

Reviewer #2, comment #2

Also the observation that unconstrained, data-driven methods find selectivity for a limited set of categories (last sentence of abstract) is not very surprising. Other data-driven methods have suggested the same, see e.g. several articles by Lashkari and Vul (NeuroImage; J. Neurophysiol.).

Our response #2.2

We note that the works cited by the authors are not entirely data-driven in the sense that the experimental stimuli used in these studies are strongly constrained by underlying hypotheses. These experiments (e.g. the work by Lashkari and Vul) further measure selectivity against a very narrow class of pre-defined coarse categories (e.g. 8 categories in the previous reference). In contrast, the network dissection procedure measures single-unit selectivity against nearly 2,000 fine-grained concepts that span not just objects, but parts of objects, colors and materials. Previous analyses have never probed selectivity at this scale. Furthermore, the selectivity analyses in this study exploit expansive naturalistic datasets in contrast to hand-curated images of a limited number of isolated objects, thereby attaining enhanced ethological validity. Our work based on the dissection approach further quantifies selectivity at a much finer-grained level, characterizing not just which images activate a particular model neuron, but what portion of the image precisely drives the high response. We note that such an approach of selectivity quantification at the pixel-level, and not the image-level, has also not been explored before.

Reviewer #2, comment #3

One aspect of the data that I find particularly striking is the observation that there is high selectivity for the preferred category even in the absence of experience with the preferred category. This is consistent with recent single-unit data in monkeys by Vinken et al. ("The neural code for 'face cells' is not face specific"). However, in contrast to the research in monkeys, the current research does not give more insight into the features that are responsible for this generalization. This finding might be present because the neural selectivity incorporates the features of the typical category images. The extent to which this is a novel and impactful datapoint depends on more information on why this is found.

Our response #2.3

By synthesizing maximally activating images for neurons in the face-deprived networks, we demonstrate that curved concentric features partially contribute to face selectivity. This is in strong alignment to prior results which have shown concentric patterns to be effective in activating the fusiform face area. In future, training models without any concentric features in the training set may disambiguate the influence and lead to stronger conclusions. Creating such a training set, however, is beyond the scope of this work. We note that our findings are further corroborated by results from Vinken et al. who show roundedness to be one of the interpretable properties predictive of face selectivity. While the spirit of analysis is similar in both these works, Vinken et al. work with networks that are still trained on faces (even though the mapping to neural data relies on non-face images). Our work on the other hand, shows that face selectivity can be gleaned even without any exposure to faces during the representation learning process.

Reviewer #2, comment #4

Many different features can be involved, which makes it difficult to test this exhaustively. I'll give one example, which will also make clear what type of analyses might be relevant. Ohayon Tsao (2012, Neuron) checked the importance of contrast features in face perception. As one part of their experiments, they worked with nonface images and analyzed how many contrast features they shared with a face template. A simple image subtraction of all non-face images with few contrast features from all non-face images with many contrast features resulted in a template that was very much face-like. This is striking because no face image was involved. I am not saying that the authors should check contrast features. However, the same analysis could already give some insights. In the nonface (face-deprived) experiment, the authors could take the (pixel-wise) average of all the nonface images that elicit strong responses and substract the average all the nonface images that elicit weak responses. How does the difference image look like? There are probably more nonlinear variants of this approach that could give insights into other features that are not defined in pixel space. There are some similarities between this approach and the methods behind Fig. 4 (which could

maybe be repeated for the deprivation experiments), but I believe that more is needed. For the methods of Fig. 4 the way that the maximally exciting inputs look like depends very much upon the regularization methods that are used.

Our response #2.4

We agree with the author that the question of what features deprived networks rely on deserves attention. Unfortunately, it is not possible to repeat the analyses in Ohayon & Tsao 2012 because the stimulus images in the natural scenes dataset consists of natural images with objects scattered in different parts of the image, unlike the Ohayon & Tsao 2012 images that have faces at the center and taking a large part of the image. Averaging over NSD images will likely wash out any systematic difference. This highlights the power of our approach that can detect the category that most activates a voxel no matter where it is in the image (See Figure 3). We further agree that the maximum exciting images definitely depends on the regularization method or priors employed. That is precisely why we chose to employ minimal regularizations to reveal the simpler feature preferences of response-optimized networks. For thorough comparison though, we now also include a new analysis in Supplementary Figure 10(shown here as well in figure 1) in which we use synthetic images optimized with naturalistic priors.

Smaller considerations:

Reviewer #2, comment #5

1) can the authors show that the results in Fig. 4 are robust to changes in the many parameters that can be varied in this approach?

Our response #2.5

The images in figure 4 have been geenrated with different random seeds in order to demosntrate robustness. The new analysis that uses GAN priors (figure 1 of this document) also demonstrates that the inferred selectivity of each region generalizes to a very different generation approach.

Reviewer #2, comment #6

2) If I understand correctly, then Fig. 6C-D show the predicted responses to 'no persons' images on the left and to 'persons' on the right. Here the model did not see persons during training, but still the analyses in Fig. 6A-B suggest that the preferred concepts were still the same (e.g., head). Based on that finding in Fig. 6A-B, I would expect that the plots in Fig. 6C-D should show an overall higher response to the 'persons' images on the right than to the 'no persons' images on the left. The change in Y-axis interval complicates this comparison, but overall this prediction does not seem to be confirmed. Can the authors explain this?

Our response #2.6

We understand the concern of the reviewer. The presence of a face in an image is not entirely indicative of the resulting activity because it still depends on the size, location, viewpoint and other features of that face since these are all complex, cluttered natural images. There is still a strong dependence between the presence of a face or of face like features (in the case of no persons images) and the activity, but this dependence is complex, and the network is rich enough to be able to learn this dependence. Thus it can still learn that face-like features will maximize the activity (under the right range of locations and sizes etc.). We have added this explanation in the *"High selectivity persists in 'face-deprived' and 'body-deprived' networks"* section of the results.

Reviewer 3 (Remarks to the Author):

Reviewer #3, comment #1

Khosla and Wehbe used deep neural network models (DNN) to analyze and interpret voxel-level activity in human higher visual areas of the 7T fMRI Natural Scenes Dataset (NSD). The models predicted quite accurately on how voxels responded to complex scenes and generalized quite rapidly to new subjects for several higher visual area ROIs, namely the fusiform face area (FFA), the extrastriate body area (EBA), the visual word form area (VWFA), and the retrosplenial cortex (RSC). More interestingly, analyzing these models provided provocative insights to interpret activity of these visual areas. The authors therefore concluded that their approach of using response-optimized DNN complements previous hypothesis-driven studies of the visual cortex, suggesting human higher visual areas may act like filters for selectively processing face, body, text, and place.

Although the manuscript describes a technology intensive work, it is in general well-written. There are several points I am hoping the authors could address to further strengthen it:

1. The selection of regions of interests (ROI) based approach. The main part of the current manuscript focuses on the FFA, EBA, VWFA and RSC. While the authors highlight that their approach was supposedly hypothesis-neutral, selecting these ROIs evidently relied heavily on hypotheses. Although I understand the last subsection of Results on pg. 16-17 and Figure 8 was more genuinely hypothesis-neutral (and I do think this part is more interesting), why did the authors not start from analyzing the whole ventral visual cortex and elaborate on this part? Alternatively, what about focusing on Brodmann areas or anatomically defined ROIs?

Our response #3.1

We understand the concern of the reviewer that our approach is not entirely hypothesis-free as we are starting from heavily studied regions, and we have provided discussion of this point in the *"Hypothesis-agnosticity reveals a limited set of categories..."* section of the Results. We initially wanted to constrain the regions that were predicted while building the end-to-end networks both to make precise inferences and because we were not sure that it was computationally feasible to train a single network for the entire visual cortex. We aimed to provide a strong case for categorical selectivity by focusing on well-studied ROIs. Testing our method on the entire ventral stream was an additional result showcasing that the categories that we can identify in this method are already discovered (including an additional category of food). It is not possible at this stage to reverse the order of the paper because many analyses such as the ones in figure 7 on the task performance of neural representations in different models (more clarifications on those analyses in response 3, comment 6) would not be useful when training the network on all the ventral stream. In particular, training ROI-specific models allows us to reveal the idiosyncratic feature preferences of different ROIs, along with their ability to support distinct tasks and functional behaviors.

Reviewer #3, comment #2

A couple of minor points relate to this issue: a) there are other face areas such as the occipital face area and the superior temporal sulcus, other scene processing area such as the parahippocampal place area, that are also well-known from previous research. The motivation of choosing the current ROIs but not other known category areas should be provided. b) Providing further method details on how exactly the ROIs were defined in the current study would be helpful.

Our response #3.2

We chose one ROI for each of the several well-studied categories (faces, places, bodies and words). We have clarified our selection in the methods section, under *"Natural Scenes Dataset"*. We have also clarified how the ROIs were obtained. In short, the authors of the NSD dataset ran the fLoc localizer by and identified for each contrast all the regions that had a positive T value. based of those regions, the FFA, EBA, VWFA and RSC along with other ROIs were identified and masks for them were constructed. The ROIs are thus constructed on an individual subject level but using a separate dataset from the one we use to train our models.

Reviewer #3, comment #3

2. Relationship of activity between different voxels. The brain is obviously not organized by voxels as

fMRI voxel is an arbitrary parcel that contains population of neurons. Based on the definition of functionally localized ROI such as FFA, EBA, VWFA and RSC, activity of voxels within an ROI should relatively correlate rather than be independent from each other. Not surprisingly, the results (Figure 5) to a large extent confirmed this point while also revealing different activity profiles between voxels within a same ROI – as the authors state that tuning differences between voxels in the same ROI were captured. However, it is not clear what this result means to understand how populations of neurons are clustered/organized based on their functions. Would it be possible the interrelationship among neurons between different voxels confound current interpretation of the models? For example, presumably if voxel size is too big, the models would fail to capture more category tuning information, but how do we know the current voxel size and shape are appropriate? On the other hand, it is well-known that category information can be decoded from multi-voxel activity patterns. Is it plausible to use response-optimized models to analyze activity patterns across multi-voxels? If so, might more category selectivity beyond face, body, text and food be found?

Our response #3.3

The issue of correspondence of neurons to voxels that the reviewer raises is an important limitation of fMRI and cannot be directly addressed by this study or other studies that only use fMRI. Nonetheless, the existence of selectivity for other categories depends on the definition of selectivity. If we consider that selectivity refers to higher activation for a category, then it would be difficult for a region (e.g. FFA) to have the same preferred category A for most voxels (i.e., most have higher activity for face) while there is another category B for which there is a distributed selectivity, because under this definition the area is most activated by A and not by B, even when looking at it as a distributed pattern. In the framework that this paper follows and provides evidence for, an area selective for a category can still have graded responses for other categories, especially when they exhibit characteristic of the preferred category. Objects from a category X can thus on average exhibit a similar pattern of activity in an area of interest (e.g. food items can have similar activations in FFA because they similarly intermediately activate the FFA "filters" even though the FFA is not selective for food), which would mean that a multi-variate pattern analysis classifier would be able to decode category X in the area of interest (e.g. decode food from FFA activation) even if this area is not selective for category X. As an analogy, one can think of a neural network detector for faces. The intermediate representations of this network for different object categories will still be similar by category because these categories share low level features, and it's very possible that one be able to decode from the internal activations of this network that the input image was a face or a car, even though the network is specialized for faces. That being said, we discuss this issue and propose to use different techniques in future work in the "Extensions" section of the discussion. We propose that future work can look at the selectivity of groups of voxels instead of individual voxels. Perhaps that the kind of selectivity that this would reveal would be a refinement of the voxel-level selectivity (e.g, one sub-group can be selective for groups of people and not just the presence of a face).

Reviewer #3, comment #4

I am also curious about relationship of voxel activity between different ROIs. If I understand correctly, through the present approach face-selective voxels were also found in the VWFA and EBA. Were text-selective voxels also found in the FFA, EBA, and RSC etc.? Are tuning functions of these voxels similar to tuning functions of the voxels selective to the conjectured preferred category of their corresponding ROIs, i.e., face-selective voxels in the FFA, text-selective voxels in the VWFA etc.? I think that exploring further on this issue and elaborating on what could be interpret from this cross-ROI analysis would be very informative.

Our response #3.4

To clarify, the ROI extraction procedure in the original dataset did not include choosing thresholds conservative enough to prevent the overlap of the identified ROIs (should explain better). Thus the ROIs that we have already have some overlaping voxels (see figure 1A). We decided not to further change the pre-selected voxels, and thus the overlap remained. It is therefore not surprising that there are face selective voxels in the VWFA and vice-versa.

Reviewer #3, comment #5

3. Reconstructed maximally exciting images. Honestly, to me they do not look like faces, texts. . . . Is

it possible to run a quick fMRI experiment on a few subjects comparing how these images may actually activate the FFA, EBA, VWFA and RSC vs. how faces, bodies, texts and places activate these ROIs?

Our response #3.5

The reason why the reconstructed maximal images don't look naturalistic is because we have not imposed a natural image prior though the use of a method like a GAN as was done previously,[5] we have clarified this in the Discussion, subsection *"Visualization of voxel selectivity with image synthesis."*. Unfortunately, running an fMRI experiment with generated images is prohibitively difficult at the time being (and we do not have access to the original subjects), but it is part of our potential major future projects. Instead, we have provided a new analysis in which we do use a GAN prior to generate images (see figure 1 in this document, also included in the supplemental materials for the paper). These images are much easier to interpret and replicate the respective selectivities of the ROIs we study.

Reviewer #3, comment #6

4. Results shown in Figure 7 are confusing or perhaps misleading. Apparently, according to these results, the RSC is better at face recognition and letter classification than layout classification; the EBA is better at face recognition and letter classification than pose prediction. Especially for the EBA, face recognition performance and letter classification performance are comparable or even better than ImageNet, but prose prediction is worse than ImageNet. Should we still call it a "body area" instead of a face area or a word form area? This is highly counterintuitive and, I think, warrants further discussions.

Our response #3.6

We note that comparing numbers across tasks is not possible since the difficulty levels of these different tasks are not equivalent, and even the baseline (chance) performance for these tasks is not the same due to the different number of classes/categories within each task. Therefore, the results from figure 7 should only be compared within tasks, and the comparative results within each task pinpoint significant functional distinctions between ROIs: the FFA-trained network is best at classifying faces, the RSA-trained network is best at classyfing scene orientation etc.

Other relatively minor issues:

Reviewer #3, comment #7

1. Many previous studies have shown idiosyncrasies of category selectivity in the ventral visual stream. For example, the FFA differs greatly across individuals as face recognition ability varies from prosopagnosia to super recognizer. Individual expertise experiences have also been argued to implicate selectivity of the FFA. Given that data from only 8 subjects were tested in the present study, external validity of the current findings warrants further discussions.

Our response #3.7

It is true that the subjects in the dataset we use were not selected based on spanning the spectrum of category specialization. Thus the results of this paper do not showcase the full variation of human category selectivity. Nonetheless, since our results are very generalizable from the four training subjects to the new training subjects with small amounts of data and since they also replicate and support major results in the literature, we believe these results to be a robust characterization of typical category selectivity . We discuss this in the Discussion section, under *"Brain response predictivity"*.

Reviewer #3, comment #8

2. Activity in the ventral visual stream is actually not fully invariant to low-level features. In addition to the well-known inversion effect of face and text processing as well as the contrast-negation effect of face recognition, high contrast and large stimulus size in general lead to greater activity in the ventral visual stream than low contrast and small stimulus size. On the other hand, intrinsic cues also activate the ventral visual stream (e.g., Cox, Meyers & Sinha, 2004). Can models in the present study speak to these complicated response properties of the ventral visual stream, or are they beyond the scope of the present study?

The extent to which response-optimized models can replicate the plethora of response properties previously reported for these regions is an interesting open question. The impressive predictive accuracy exhibited

by these models, along with their robust generalization to unfamiliar synthetic stimuli and subjects, provides a promising indication that these models might effectively replicate the previously known low-level feature preferences of these ROIs, while also generating novel hypotheses regarding their response tuning. While thorough analyses replicating the full set of documented low-level preferences of these regions is beyond the scope of the present study, we do include the discussion of this potential application of our models in the *"Extensions"* subsection of the Discussion.

Reviewer #3, comment #9

3. While the VWFA is normally only found in the left hemisphere, the FFA is often found in both hemispheres but larger in the right than in the left. It is not clear whether the present study noted the hemispherical lateralization, or simply pooled data from both hemispheres.

Our response #3.9

The present study pools the FFA voxels from both hemisphere. Note however that the FFA was functionally localized per subject, so if it is smaller in the left hemisphere, less voxels would have been selected by the functional localizer. The hemispherical lateralization was indeed notable after applying the functional localizers. On average, across the four NSD participants, 68% of the localized VWFA voxels belonged to the left hemisphere. The right lateralization of FFA was also noticeable: 58% of the localized voxels were located within the right hemisphere. We report these lateralization effects in the Methods *"Natural Scenes Dataset"* section of our manuscript.

References

[1] Katja Seeliger, Luca Ambrogioni, Yağmur Güçlütürk, Leonieke M van den Bulk, Umut Güçlü, and MAJ van Gerven. End-to-end neural system identification with neural information flow. *PLOS Computational Biology*, 17(2):e1008558, 2021.

[2] Ghislain St-Yves, Emily J. Allen, Yihan Wu, Kendrick Kay, and Thomas Naselaris. Brain-optimized neural networks learn non-hierarchical models of representation in human visual cortex. *bioRxiv*, 2022.

[3] Meenakshi Khosla, Keith Jamison, Amy Kuceyeski, and Mert Sabuncu. Characterizing the ventral visual stream with response-optimized neural encoding models. *Advances in Neural Information Processing Systems*, 35:9389–9402, 2022.

[4] N Apurva Ratan Murty, Pouya Bashivan, Alex Abate, James J DiCarlo, and Nancy Kanwisher. Computational models of category-selective brain regions enable high-throughput tests of selectivity. *Nature communications*, 12(1):5540, 2021.

[5] Zijin Gu, Keith Wakefield Jamison, Meenakshi Khosla, Emily J Allen, Yihan Wu, Thomas Naselaris, Kendrick Kay, Mert R Sabuncu, and Amy Kuceyeski. Neurogen: activation optimized image synthesis for discovery neuroscience. *NeuroImage*, 247:118812, 2022.

[6] Andrew Luo, Maggie Henderson, Leila Wehbe, and Michael Tarr. Brain diffusion for visual exploration: Cortical discovery using large scale generative models. *Advances in Neural Information Processing Systems*, 36, 2023.

[7] Chris Olah, Arvind Satyanarayan, Ian Johnson, Shan Carter, Ludwig Schubert, Katherine Ye, and Alexander Mordvintsev. The building blocks of interpretability. *Distill*, 3(3):e10, 2018.

[8] David H Hubel, Torsten N Wiesel, and Michael P Stryker. Anatomical demonstration of orientation columns in macaque monkey. *Journal of Comparative Neurology*, 177(3):361–379, 1978.

[9] RB Tootell and Susan L Hamilton. Functional anatomy of the second visual area (v2) in the macaque. *Journal of Neuroscience*, 9(8):2620–2644, 1989.

[10] Xiaomin Yue, Irene S Pourladian, Roger BH Tootell, and Leslie G Ungerleider. Curvature-processing network in macaque visual cortex. *Proceedings of the National Academy of Sciences*, 111(33):E3467–E3475, 2014.

[11] Alexander S Ecker, Fabian H Sinz, Emmanouil Froudarakis, Paul G Fahey, Santiago A Cadena, Edgar Y Walker, Erick Cobos, Jacob Reimer, Andreas S Tolias, and Matthias Bethge. A rotation-equivariant convolutional neural network model of primary visual cortex. *arXiv preprint arXiv:1809.10504*, 2018.

[12] J Swaroop Guntupalli, Michael Hanke, Yaroslav O Halchenko, Andrew C Connolly, Peter J Ramadge, and James V Haxby. A model of representational spaces in human cortex. *Cerebral cortex*, 26(6):2919–2934, 2016.

[13] David A Klindt, Alexander S Ecker, Thomas Euler, and Matthias Bethge. Neural system identification for large populations separating what and where. In *Proceedings of the 31st International Conference on Neural Information Processing Systems*, pages 3509–3519, 2017.

[14] David Bau, Bolei Zhou, Aditya Khosla, Aude Oliva, and Antonio Torralba. Network dissection: Quantifying interpretability of deep visual representations. In *Proceedings of the IEEE conference on computer vision and pattern recognition*, pages 6541–6549, 2017.

[15] David Bau, Jun-Yan Zhu, Hendrik Strobelt, Agata Lapedriza, Bolei Zhou, and Antonio Torralba. Understanding the role of individual units in a deep neural network. *Proceedings of the National Academy of Sciences*, 117(48):30071–30078, 2020.

[16] Doris Y Tsao, Winrich A Freiwald, Roger BH Tootell, and Margaret S Livingstone. A cortical region consisting entirely of face-selective cells. *Science*, 311(5761):670–674, 2006.

Reply to the Reviewers

"Higher visual areas act like domain-general filters with strong selectivity and functional specialization"
Meenakshi Khosla and Leila Wehbe
Nature Communication

Dear Editor,
We thank you for arranging the review and giving us an opportunity to improve our manuscript. Please find below the responses to the reviewer comments.

REVIEWER COMMENTS

Reviewer 1 (Remarks to the Author):

Reviewer #1, comment #1

The authors have thoroughly addressed all of the points raised in my review, and I am satisfied with the revisions made. The manuscript is now suitable for publication. I would also like to thank the authors for their detailed and thoughtful responses in the rebuttal letter.

Reviewer 1 (Remarks on code availability):

The code is clearly written and commented. The authors also provide demos with limited data that can easily be used. A README file and examples in Jupyter notebooks are also provided. The code can be used for understanding what has been done and for reproduction, and become the basis for similar projects.

Our response #1.1

We thank the reviewer for their positive assessment.

Reviewer 2 (Remarks to the Author):

Reviewer #2, comment #1

I thank the authors for the thorough discussion of the points that I brought up. I have sympathy for many of their points. This is an interesting study with a lot of innovation at the level of using brain data to train computational models.

Our response #2.1

We thank the reviewer for their positive comments.

Reviewer #2, comment #2

Nevertheless, I am concerned that novelty for the understanding of the neural basis of object recognition and categorization is still not well characterized and several neuroscience-related findings in the literature are mostly ignored despite being very similar. Maybe my previous comments were at times too conceptual and as such not always probing for specific actions, so I'll give two very specific suggestions that are already implied by my previous review.

First, a better discussion of the differences with Ratan Murty et al (2021) is needed. This study already showed the relevance of computational models for high-throughput tests (and alternatives for fMRI experiments), and many of the messages are the same. The submitting authors point to the use of task-trained

models in that study as the major difference with their own training on brain data. This is not as absolute though. Ratan Murty also trained the classifier with brain data, with the difference being in where the input features came from. However, in addition, Ratan Murty showed good performance with relatively generic feature layers (late convolutional layers), and for models with very different task objectives some of which are very remote from the investigated categories (e.g., using training on scenes with Places365 to give features for a classifier trained on FFA responses). So as soon as high-level features are present, it works. Findings like Murty's make me less surprised by the findings in the submitted manuscript. I can see the interest from a modelling perspective (look, we seem to have enough data to train on the brain data per se!), but the novelty from a neuroscience and cognitive point of view is less prominent to me once I consider what Murty et al found.

Our response #2.2

We agree with the reviewer that a better comparison with Murty et al (2021)[1] is required. Our work differs in several key ways—both methodologically and scientifically—which we outline below.

Methodological differences:

- Training directly from neural activity vs. using task-trained features: Our models are trained end-to-end exclusively on voxel-level fMRI data, so high-level features (for example, face detectors) emerge solely from the objective of reproducing the brain's responses. In contrast, Murty et al. rely on networks pre-trained on large-scale object or scene classification tasks (e.g., Places365), a dataset comparable in scale to ImageNet (∼1.8 million images) that is known to contain numerous category-selective units (including those selective for faces, see Bau et al.(2017)[2]). In these task-driven networks, rich semantic signals are already embedded in the feature layers, enabling them to predict responses by leveraging pre-existing category-level information. Because our models must "earn" complex category-selective representations solely from brain data—without any explicit labels—they provide a more stringent test of the underlying neural computations.
- Voxel-level modeling vs. region-level aggregation: While Murty et al. demonstrate success using aggregate, region-level signals, our approach models brain activity at the voxel level. This fine-grained modeling allows us to capture subtle variations in neural tuning within a given region. For example, our method can distinguish between face-selective units that might otherwise be grouped under a broader label (such as those inadvertently included in the VWFA due to liberal thresholds), thus revealing heterogeneity within the region that region-level aggregation would obscure.
- Automated Network Dissection with a large concept dictionary: We apply an automated network dissection procedure using a systematic library of nearly 2000 concepts—including high-level objects, object parts (e.g., eyes, ears), colors, and textures—to directly quantify and verbalize the selectivity of each model unit. This procedure not only identifies which images drive a voxel's response but also pinpoints the precise segments within an image (e.g., determining if face selectivity is driven primarily by the eyes). In contrast, Murty et al. primarily focus on image-level responses, which limits the ability to resolve the specific features underlying selectivity.

Scientific differences:

- Revealing a limited set of emergent categories: By training a model on the entire ventral visual pathway, we demonstrate that only a few categories—namely faces, text, and bodies—consistently emerge as driving strong selectivity. This finding challenges the notion of a vast, undiscovered set of category-selective regions, suggesting instead that the principal semantic dimensions in high-level visual cortex may already have been discovered.
- Evidence for functional specialization: Our region-specific models not only predict neural activity with high accuracy but also show distinct functional specializations in downstream tasks. For instance, representations derived from the FFA outperform others in face recognition tasks, whereas those from

the RSC excel in spatial layout estimation. In contrast, previous studies using task-trained or region-level models—employing the same feature space across regions—could not dissociate these functional capabilities.

- Visual Deprivation Experiments: Our "visual diet" experiments reveal that hallmark responses of certain brain regions can emerge even without direct exposure to their putative category (e.g., faces or bodies). When models are trained on stimulus sets that intentionally omit these categories, they still develop selective responses. This suggests that the selectivity in these cortical regions is driven by general visual features that may also be present in other stimuli, rather than by direct category exposure alone.

We have added the following paragraph to our discussion (see lines 742-751):

> *"Our findings extend prior work (e.g., Murty et al., 2021) by showing that purely response-optimized models—trained exclusively on fMRI signals at the voxel level—can learn high-level category detectors without any explicit category labels. Moreover, by dissecting trained networks with a large-scale concept dictionary, we pinpoint the exact objects and object parts driving each voxel's response. This methodology not only reveals that category-selective responses can emerge from the demands of predicting neural activity alone (even in the absence of direct training on those specific categories), but also highlights the existence of unique functional capacities in different ventral regions—illustrated by their specialized performance on tasks such as face recognition, body posture classification, or spatial layout estimation."*

Reviewer #2, comment #3

Second, the authors should refer to the work of Vinken et al. (2023, Science Advances) that came to very similar conclusions in terms of higher visual responses acting like "domain-general filters". The similarity in the main conclusions is striking. A deep discussion of this convergence is needed. Given that I mentioned this paper very clearly in my previous review, and the authors also cite that paper when replying to my comments, I am surprised that the Vinken et al paper is even not mentioned at all in the revised version. (And no, I am not an author of that paper.)

Our response #2.3

We thank the reviewer for bringing this issue again. We have modified the text in the following manner. Also, not mentionning the Viken et al. paper was an oversight that we have also corrected. We have included the following text in our discussion (see lines 662-672 in the revised manuscript):

> *"These results build on the work of Vinken et al. [3] Both studies converge on the idea that face selectivity does not rely on features exclusive to faces but instead arises from domain-general visual representations (with our findings extending to bodies as well). However, our approaches differ substantially. Vinken et al. rely on the observation that face-selective sites show reliable responses to non-face images and a common encoding axis in object-trained networks can predict face selectivity—networks that are, nonetheless, trained on datasets including faces, potentially embedding face-selective units. In contrast, our selective deprivation experiments remove face (or animate) stimuli entirely from the training set. This design eliminates the potential confound of prior face exposure and directly tests whether category-specific visual experience is necessary to account for responses in corresponding category-selective regions."*

Reviewer #2, comment #4

As another lingering concern, an important part of the paper concerns the training with and without the preferred category being present. Figure 6 and the summary in the manuscript emphasize how well this works, but the summaries should retain the important finding that the correspondence with the preferred concept is apparently going down from 0.13 to 0.08, so halfway to the threshold of 0.04 that the authors set themselves throughout the paper. This is a major reduction of selectivity, and should be mentioned in e.g. Abstract and Discussion. At the very very least, the relevant sentence in the abstract should read "this

selectivity is MOSTLY maintained", and summaries in e.g. Discussion should mention this reduction. Also, in the figures, it seems important to plot the IoU to the preferred category of the face/body-deprived models next to the IoU of the non-deprived models.

Our response #2.4

Following the suggestions of the reviewer, we made modifications to the abstract (line 17), introduction (line 99), result section (lines 377-378) and discussion (lines 645-647) to highlight further that the selectivity is reduced. Note that we already had mentioned this fact in the result section, were we had written (lines 373-377):

> *"The training diet deprivation did decrease the agreement of model voxels with their respective preferred category (e.g., in FFA, the median IoU with 'head' dropped from $\sim$ 0.13 in the non-deprived network to $\sim$ 0.08 in the deprived network), indicating a slightly reduced selectivity (Fig. 6).".*

We feel it will lead to overcrowding to add the sub-figures again to Figure 6 and hope that due to our referencing the previous results multiple times the readers will look again at the previous relevant figures..

Reviewer #2, comment #5

Note also that the stimulus set does not include images that are optimized for ROIs like FFA and EBA. The example "most activating images" in Fig. 3 make this very obvious. FFA is most activated by close-up images of faces, not by the images shown here (also Ratan Murty showed this very clearly). As a consequence, the fitted models might miss important aspects of the selectivity of such ROIs, and it is very much conceivable that these are the selectivity properties that are most strongly affected by the presence/absence of faces in the image set. Or, to put it in the context of the text that the authors added on p. 15, would you expect a particularly high response in your FFA model for close-up faces, and would you predict this to be the same with face deprivation? Such out-of-distribution tests seem very important here, considering that they implicate the sort of images that other studies have shown to be optimal for FFA. The lack of such images in your stimulus set is worth discussing.

Our response #2.5

We thank the reviewer for raising this point. We realize now that we have not made it clear that the segmentation map examples in Figure 3 are generated using the top images from the Places265 dataset employed in our network dissection procedure, and not from the NSD stimulus set. In fact, the original NSD dataset does include many images with close-up faces that are optimal for ROIs such as the FFA. To further support this, Figure 1 below shows 20 images—selected from the NSD training and test sets—that produce the highest mean predicted response for the response-optimized FFA model. These images, with the dissection procedure applied to highlight the contributing regions, clearly demonstrate the presence of close-up faces. Moreover, the presence of such images in NSD is corroborated by samples reported in previous studies, including Khosla et al. (2022)[4] and Luo et al (2023)[5] (see Figs. 2B and 4 respectively in these papers).

We clarified this in the description of the image and the methods section (lines 217-220) which reads:

> *"The technique measures the degree of alignment between a voxel's response properties and an extensive visual concept dictionary, spanning objects and fine-grained visual concepts like parts of objects, colors, materials, and textures. This alignment is estimated on an independent, large-scale natural image database called the Places365 dataset comprising a diverse set of real-world environments (indoor, urban, and natural). These images were not seen by the subjects during the experiment."*

Reviewer #2, comment #6

[editorial note: figure redacted]

Figure 1: Top 20 images producing the highest mean predicted response for the response-optimized model of FFA. The images are drawn from the training (top) and test (bottom) set of NSD. Activation of a single voxel (converted to a 1x1 convolutional filter) in response to an input image is visualized as the region in the input space that elicits the highest (top 1% quantile level) activation in the corresponding filter output. For every image, a random voxel was selected from the FFA model for this dissection procedure.

Finally, it now occurs to me that the authors selected ROIs/domains for the deprivation test. I can see why you would do that for RSC given that the preferred category is probably difficult to remove completely from natural images, but I don't find good arguments for why one would not include VWFA in this test. There were not so many billboards in the images according to the authors, so this test should be very easy to implement? Or did the authors try and it failed?

Our response #2.6

We appreciate the reviewer's comment. We limited the deprivation experiment to faces and bodies because the NSD images are sourced from MS-COCO, which offers detailed pixel-level annotation masks for the 'person' and broader 'animate' categories. These rich annotations enable us to confidently exclude any image that contains faces or bodies—even if they occupy only a small portion of the image. In contrast, comparable dense annotations for 'text' are not available, making it impossible to ensure that all text has been completely removed from the dataset (which would be relevant for VWFA analyses).

Reviewer 3 (Remarks to the Author):

Reviewer #3, comment #1

First of all, I would like to thank the authors to take such a long time to address my previous concerns. I think the manuscript has been strengthened to a large extent. However, I remain concerned about the following points.

*3.1 I understand that to train a network for the entire visual cortex would not be possible. As a matter of fact, there has never been any clear definition boundary of where exactly 'visual cortex' may include. However, the current overall framing of the manuscript heavily relies on and indeed still stresses on 'hypothesis-neutral'. And I agree that the manuscript should have been a lot stronger if the approach could really be hypothesis-neutral. As I suggested previously, using anatomically defined ROIs would make the current study a lot closer to a hypothesis-neutral approach. Note that the so called *ventral* visual cortex involves a mixture of anatomical and functional concepts, therefore may not be ideal. But subsampling subsections of the inferior temporal cortex that can be anatomically defined should be feasible and more like hypothesis-neutral for functional category selectivity.*

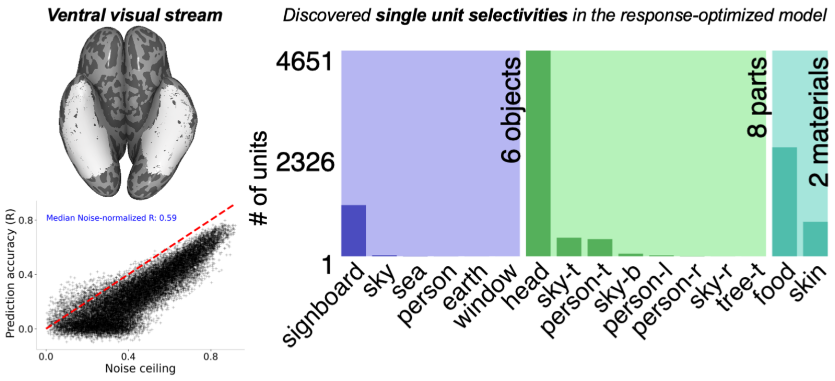

r

Figure 2: Dissection (right) of a response-optimized model trained to predict responses across the entire ventral visual stream (left, shown for one participant). Bottom left plot shows the un-normalized prediction accuracy (R) of every ventral visual stream voxel against the corresponding noise ceiling. Noise-normalized prediction accuracy is reported in the inset.

Our response #3.1

To clarify, the analysis in Figure 8 of our previous submission did not rely on functionally defined ROIs but was instead based entirely on an anatomical atlas. We extracted voxels corresponding to the ventral visual stream from the Natural Scenes Dataset using anatomically defined ROIs, which were manually delineated for each subject. Specifically, the selected ROI—a subsection of the inferior temporal cortex defined solely by anatomical landmarks (following the anterior lingual sulcus, anterior lingual gyrus, inferior temporal sulcus, and the midpoint of the occipital temporal sulcus)—comprises approximately 7,000–9,000 voxels per subject (totaling 28,910 voxels across all subjects) and is shown in Figure 2 below. This approach directly addresses the reviewer's concern by providing a hypothesis-neutral analysis based on anatomical definitions.

The full methodological details can be found in lines 1107-1123, which state:

> *"**Training a model on the entire ventral visual cortex** To test whether category selectivities may be uncovered with an impartial approach that does not require ROI selection or localization, we trained a single response-optimized model on responses from the entire ventral visual stream across all subjects. The model employed the same architecture as the ROI-specific models and was trained following the same procedure as employed for ROI-specific models and described above. The ventral visual stream voxels in every subject were extracted using the streams atlas released with the Natural Scenes Dataset.*[6] *Briefly, this ROI collection reflects large-scale divisions of the visual cortex into primary visual cortex and intermediate and high-level ventral, lateral and dorsal visual areas. These were manually drawn by NSD curators for each subject after accounting for the voxel-level reliability metrics. We extracted the ROI mask corresponding to the higher-level ventral stream label. This ROI was drawn to follow the anterior lingual sulcus (ALS), including the anterior lingual gyrus (ALG) on its inferior border and to follow the inferior lip of the inferior temporal sulcus (ITS) on its superior border. The anterior border was drawn based on the midpoint of the occipital temporal sulcus (OTS). As shown in Figure 2, it is very broad (∼7,000-9,000 voxels per subject), yielding a total of 28,910 voxels across all 4 NSD subjects."*

We also include below the subsection that details these results (lines 494-524):

> *"**Hypothesis-agnosticity reveals a limited set of categories for which selective responses are observed in the ventral visual cortex** We note that our approach, thus far, was hypothesis-neutral in the sense of model construction; i.e., the models were unconstrained by any particular hypothesis, rather they were largely informed by the data. While some of our architecture choices were informed by* a-priori *knowledge of what could be important to model the primate visual system in general (convolutional structure, equivariant filters), these choices remain neutral with respect to the selectivities of the high-level ROIs we study. Nonetheless, the*

selection of regions itself was biased by apriori knowledge and their localization was guided by independent functional data measuring the brain responses to different categories of stimuli. We presented an approach to determine the selectivity of these regions and characterize their function in an impartial computational manner. The emergence of category-selective units in response-optimized models unbidden provided emphatic evidence for category-selectivity of these regions and suggested that these regions not only respond to low-level features most correlated with the preferred category but to the high-level category per se. Here, we take a step further and ask: can we discover category selectivities with an impartial approach that does not require ROI selection or localization? To answer this question, we performed a retrospective analysis: we trained a response-optimized model on responses from the entire ventral visual cortex (28,910 voxels) across all subjects. The resulting model is able capture the structure of ventral visual stream responses, as evidenced by a reasonably high response prediction accuracy (median noise-normalized R∼59%). We then applied the network dissection procedure on units within the optimized model. The results, shown in Figure 2, highlight the categories previously believed to elicit selective responses, namely heads (or, faces), signboards (or text) and skin, which reflects selectivity for the 'bodies' category. A small number of units appear selective for elements such as sky and window, which can be attributed to processing scenes. Moreover, we also observe single-unit selectivities for the 'food' category, for which neural selectivity was also reported in recent works,[4, 7, 8] validating our data-driven approach. The non-existence of units aligned with any of the thousands of other semantic concepts beyond these categories in the dissection concept dictionary (e.g. car, door, chair, plastic etc.) also serves as evidence against the existence of selectivity for those categories of stimuli in the ventral visual cortex."

Reviewer #3, comment #2

3.5 Using GAN is a good idea! And I agree the new set of generated images are much easier to interpret. However, it is not clear what brain mechanisms may correspond to GANs that can be added on to the current part of visual processing. I think the authors do make a decent stab of explaining why this greatly helps the visualization of voxel selectivity (p.21-22) but equally I think that potential readers may still get confused on how the brain could incorporate generative mechanisms or GAN-based priors versus diffusion model priors for visual category selectivity. Elaborating theoretical possibilities or flag this as a theoretical limitation in the Discussion section would be greatly helpful.

Our response #3.2

Thanks for this comment. To clarify, our use of GAN-based priors is solely a methodological tool to enhance the interpretability of voxel selectivity, not an attempt to model actual generative brain processes. In fact, as shown in Figure 4 in our main paper, our analysis using simpler regularizers—which do not imply any neurobiological generative mechanism—produces results that are loosely consistent with those obtained using the GAN-based prior (albeit less interpretable). This demonstrates that our main scientific conclusions are robust across different forms of regularization. It is also possible to do this analysis with a diffusion prior instead of a GAN prior, and in concurrent work it has been shown that it leads to similar scientific conclusions.[5]

In the revised manuscript, we include an expanded discussion that clearly distinguishes the methodological benefits of using GAN-based priors from any claim that they mirror the brain's generative processes. (see lines 615-618 in the Discussion section). It reads:

> *"It is important to clarify that our use of GAN-based priors is solely a methodological strategy for enhancing the visualization of voxel selectivity and does not imply that the brain employs similar generative mechanisms."*

We further discuss this in lines 626-629:

We believe that this effectively addresses the reviewer's concern and helps prevent any misinterpretation regarding the neurobiological implications of our generative approach. *"Visualization*

with a natural image prior may not always be truly faithful to the model or to the brain, as the natural image prior is learned from a distribution of images in a large dataset, and does not correspond to a process that the brain is engaged in. In other words, the learned generative distribution doesn't map to a generative process that the brain is doing, and instead is simply used to facilitate interpretation"

We believe that this effectively addresses the reviewer's concern and helps prevent any misinterpretation regarding the neurobiological implications of our generative approach.

Reviewer #3, comment #3

3.6 I understand that classification accuracy should not be compared across tasks. However, Figure 7 is still confusing. Comparing within the same task, performances of the other three ROIs than the category selective ROI often vary, sometimes apparently significantly differ (e.g., for letter classification and for body pose classification). Use ImageNet performances (blue triangles) as a benchmark to compare within each task may be a good idea. But this should be more explicitly described in the manuscript. Plus, text descriptions (p. 17) do not match Figure 7. The authors should proofread the manuscript more carefully.

Our response #3.3

We thank the reviewer for this comment. We have updated the organization Figure 7 to make it more clear and we have revised the text to emphasize the results and comparison with the ImageNet-trained network's baseline, and we revisited the description in section "Models reveal key functional distinctions between different regions" to align with the figure.

The following texts have been added to make the ImageNet comparisons more explicit in our revised manuscript:

- Explicit Benchmark Mention:
  "In this and every subsequent task, we include the performance of a general-purpose representation from an ImageNet-trained network (denoted as blue triangles in Figure 7) as a benchmark." (lines 408-410)
- Clarification on Task Comparisons:
  "It is important to note that classification accuracy is inherently task-specific, so direct comparisons across tasks are not meaningful; however, within each task, the ImageNet baseline provides a meaningful benchmark for contextualizing the performance of our ROI-specific models." (lines 452-455)
- Task-Specific Discussion:
  - Face Discrimination:
    "The resulting FFA predictions outperform the predictions from all other ROI models (discrimination score of 85% compared to 79–80%), even outperforming the highly transferable representation of ImageNet-trained networks[9] at 78% (see Figure 7B)." (lines 414-417)
  - Letter Classification:
    "The VWFA response-optimized model consistently outperforms the remaining response-optimized models and the benchmark set by the ImageNet-trained network (see Figure 7C), providing computational support for the ability of VWFA responses to enable letter perception." (lines 424-427)
  - Body Pose Classification:
    "On this pose-discrimination task, we find that the EBA model consistently performs on par with or exceeds the performance of the remaining ROI models (see Figure 7D). Here, however, we observe a gap between the performance of the best response-optimized model and the ImageNet-trained task-optimized model, possibly due to the richer variation of different body postures in the ImageNet dataset compared to NSD stimuli, which may allow efficient transfer to this task, or to the particular challenges that the body pose estimation task presents." (lines 432-438)
  - Room Layout Estimation:
    "The RSC predictions outperform the other ROI models. They perform just as well as the ImageNet-trained representations in solving this task, even though the RSC network was trained with ∼35,000 images and their associated brain responses, in contrast to the ImageNet network's training size of a million images." (lines 446-450)

References

[1] N Apurva Ratan Murty, Pouya Bashivan, Alex Abate, James J DiCarlo, and Nancy Kanwisher. Computational models of category-selective brain regions enable high-throughput tests of selectivity. *Nature communications*, 12(1):5540, 2021.

[2] David Bau, Bolei Zhou, Aditya Khosla, Aude Oliva, and Antonio Torralba. Network dissection: Quantifying interpretability of deep visual representations. In *Proceedings of the IEEE conference on computer vision and pattern recognition*, pages 6541–6549, 2017.

[3] Kasper Vinken, Jacob S Prince, Talia Konkle, and Margaret S Livingstone. The neural code for "face cells" is not face-specific. *Science Advances*, 9(35):eadg1736, 2023.

[4] Meenakshi Khosla, N Apurva Ratan Murty, and Nancy Kanwisher. A highly selective response to food in human visual cortex revealed by hypothesis-free voxel decomposition. *Current Biology*, 2022.

[5] Andrew Luo, Maggie Henderson, Leila Wehbe, and Michael Tarr. Brain diffusion for visual exploration: Cortical discovery using large scale generative models. *Advances in Neural Information Processing Systems*, 36:75740–75781, 2023.

[6] Emily J Allen, Ghislain St-Yves, Yihan Wu, Jesse L Breedlove, Jacob S Prince, Logan T Dowdle, Matthias Nau, Brad Caron, Franco Pestilli, Ian Charest, et al. A massive 7t fmri dataset to bridge cognitive neuroscience and artificial intelligence. *Nature neuroscience*, 25(1):116–126, 2022.

[7] Nidhi Jain, Aria Wang, Margaret M Henderson, Ruogu Lin, Jacob S Prince, Michael J Tarr, and Leila Wehbe. Food for thought: selectivity for food in human ventral visual cortex. *bioRxiv*, 2022.

[8] Ian Morgan Leo Pennock, Chris Racey, Emily Jean Allen, Yihan Wu, Thomas Naselaris, Kendrick Kay, Anna Franklin, and Jenny Bosten. Color-biased regions in the ventral visual pathway are food-selective. *bioRxiv*, 2022.

[9] Minyoung Huh, Pulkit Agrawal, and Alexei A Efros. What makes imagenet good for transfer learning? *arXiv preprint arXiv:1608.08614*, 2016.

We thank the reviewer for emphasizing the importance of the whole-ventral-stream analysis (Fig. 8). We fully agree that this experiment usefully demonstrates that category-selective detectors emerge even when *no anatomical prior* is supplied. Our decision to keep Fig. 8 compact, however, was deliberate. The central claim of the manuscript is *not* merely that hypothesis-neutral training yields face/word/body detectors, but that *separate response-optimized models expose functional distinctions between ROIs* by selectively transferring to face-ID, body-pose, letter, and layout tasks. A single pan-ventral model, by construction, conflates those areas and therefore cannot address whether FFA, VWFA, EBA, and RSC differ in representational *function*. It would not be able to let us perform the analyses in figure 7. Highlighting the ROI-specific models allows us to test—and confirm—long-standing functional accounts that could not be evaluated with one combined model. We hope this clarifies why the manuscript concentrates on ROI-specific models while still acknowledging the importance of the whole-ventral analysis.

Response to reviewers

Reviewer #2 (Remarks to the Author):

The authors have addressed all my remaining suggestions. I congratulate them with this very nice paper.

We thank the reviewer for their congratulations and their contributions to the paper.

Reviewer #3 (Remarks to the Author):

Again, first of all I thank the authors for taking so long to address our concerns. But I have to say this process has taken way too long. I had forgotten many details and had been quite confused with how the authors addressed my second round comments, until I found my first round comments to the original submission over two years ago.

In my first round comments, I highlighted the results shown in Figure 8 (Figure 2 in the response letter of this round), and suggested the authors to elaborate on this part of their study. I suggested this because, as I pointed out in the first round, this part was genuinely hypothesis-neutral and thus most interesting. Unfortunately, the authors apparently did not take the suggestion as an opportunity to strengthen the paper in that round of their revision. They however highlighted Figure 8 only in the response letter of this round to address my concerns. Again, my suggestion is to work on this part as potentially stronger evidence to support their claim of hypothesis-neutral. It should not be about convincing me or anyone as a reviewer. I just think that highlighting and elaborating on this part will strengthen the paper and benefit potential readers and thus making the paper potentially more impactful.

We thank the reviewer for their contributions to the paper and apologize for the long delay. We understand the perspective of the reviewer of focusing on figure 8 as truly hypothesis-neutral. However, the paper presents multiple points and some of them require the setup we have provided. Mainly, the main argument that brain-response optimization confers functional specialization (figure 7) can only work in the setup we have presented.
